# DABCO Derived Nitrogen-Doped Carbon Nanotubes for Oxygen Reduction Reaction (ORR) and Removal of Hexavalent Chromium from Contaminated Water

**DOI:** 10.3390/ma14112871

**Published:** 2021-05-27

**Authors:** Vadahanambi Sridhar, Hyun Park

**Affiliations:** 1 Global Core Research Centre for Ships and Offshore Plants (GCRC-SOP), Pusan National University, Busan 46241, Korea; 2 Department of Naval Architecture and Ocean Engineering, Pusan National University, Busan 46241, Korea

**Keywords:** reduced graphene oxide, nitrogen-doped carbon nanotubes, oxygen reduction reaction (ORR), palladium, chromium removal

## Abstract

Though chemically-derived reduced graphene oxide (CDG) from graphite oxide (GO) precursors is a widely practiced procedure for the large-scale production of graphene, the quality and quantity of thus obtained CDG is dependent on the reduction strategy used. In this work, we report an all-solid-state, residue-free, microwave process for the reduction of graphene oxide and subsequent growth of carbon nanotube ‘separators’ from a single precursor, namely DABCO (1,4-diazabicyclo[2.2.2]octane). The utility of our newly developed technique in efficiently and effectively reducing graphene oxide and in growing nitrogen-doped carbon nanotubes via catalysts like palladium and iron into unique mesoporous, 3-D hierarchical carbon nanostructures is demonstrated. The applicability of the thus obtained palladium embedded in Pd@NCNT-rGO nanoarchitectures for the oxygen reduction reaction (ORR) is investigated. When carbon fiber (CF) was used as the substrate, three-dimensional Fe@NCNT-CF were obtained, whose capability as versatile adsorbents for hexavalent chromium ion removal from contaminated waters was also demonstrated.

## 1. Introduction

The depletion of fossil fuels and environmental effects due to its combustion have necessitated the need for clean, green and energy-efficient fuels. Amongst the many alternatives, fuel cells have emerged as effective and environmentally friendly power generation devices due to their high energy densities. However, a major hurdle restricting their large-scale deployment is the absence of reliable, low-cost and efficient catalysts for the oxygen reduction reaction (ORR). Until now, Pt/C electrodes constituting nanosized platinum particles dispersed in a conductive carbon matrix have been widely recognized as effective ORR catalysts but suffer from inherent drawbacks, like ever-increasing prices and the deactivation of the catalyst during prolonged cycling, which necessitates the need for cheap, effective and long-lasting non-Pt-based catalysts. Amongst the various alternatives, palladium (Pd)-based nanostructures have attracted wide attention from researchers as ORR catalysts in fuel cells. When compared to commercially available Pt-based catalysts, the nanostructured Pd catalysts are less expensive and widely available and are capable of exhibiting a high ORR activity in alkaline medium [1,2]. However due to their low electrical conductivity, pristine Pd nanoparticles are seldom used in ORR reactions, and for most practical purposes they have to be hybridized with conductive materials like carbon black [3], graphene [4], carbon nanotubes (CNTs) [5], etc. This hybridization of Pd with carbonaceous materials can be broadly divided into in-situ or ex-situ techniques. Amongst these, the ex-situ technique is widely practiced, wherein presynthesized Pd nanoparticles are hybridized with carbonaceous substrates. However, in order to obtain a good anchoring and effective hybridization, the carbonaceous substrates must be functionalized, which usually involves embedding oxygen moieties, thereby reducing the inherent conductivity. The in-situ method involves a reduction of palladium precursors either by sol-gel [6] or by hydrothermal [7] or electrochemical methods in graphene and CNT dispersions to obtain Pd-carbon hybrids. Despite the ease and straightforward nature of the in-situ technique, it suffers from disadvantages of the Ostawald ripening-induced aggregation of Pd nanoparticles and the necessity of using expensive surfactants if one wants to mitigate this aggregation. Therefore, in order to overcome these practical difficulties associated with both these techniques, there is a necessity for the development of a single-step procedure for the synthesis of Pd nanoparticles anchored within 3-D conductive carbon substrates.

Hexavalent chromium (Cr(VI)) is an obnoxious byproduct of the electroplating, metal finishing and leather tanning industries and must, due to its carcinogenicity and toxicity, be removed from waste water below the tolerance limit of 0.1 mgL^−1^, the recommended limit of USEPA (United States Environmental Protection Agency). Until now, a wide range of techniques like adsorption [8], chemical precipitation [9], electro-deposition [10], filtration by membranes [11], ion-exchange [12], etc. have been investigated for the effective removal of Cr(VI) from contaminated waters, of which adsorption is widely practiced due to its good efficiency and due to the availability of low-cost adsorbents. Until now, carbon materials like activated carbon [13], carbon fiber [14], carbon black [15], biomass derived carbon [16], graphene [17], etc. have been used, but the usage of carbon nanotube/carbon fiber hybrids for the removal of hexavalent chromium has never been reported.

Herein, we report the synthesis of palladium nanoparticles dispersed in 3-D mesoporous nanoarchitectures consisting of nitrogen-doped carbon nanotubes vertically anchored on a reduced graphene substrate (Pd@NCNT-rGO) by a simple microwave method using DABCO as the precursor for the concurrent reduction and growth of nitrogen-doped carbon nanotubes. When applied as electrodes in the ORR reaction, our Pd@NCNT-rGO synthesized by the one-step procedure is capable of delivering an excellent ORR activity when compared to expensive commercial Pd/C. The applicability of our newly discovered DABCO precursor for the synthesis of NCNT with other catalysts, like iron, and on microwave susceptible surfaces, like carbon fibers, is demonstrated, and the utility of Fe@NCNT-CF for the removal of hexavalent chromium from contaminated water is also demonstrated. In this manuscript, the utility of Fe@NCNT-CF as efficient adsorbents for the removal of Cr(VI) is reported for the first time. Our newly developed technique is not only fast, but it can also be scaled up to the gram-scale production of functionalized carbon nanohybrids for energy and environmental applications.

## 2. Experimental Section

### 2.1. Materials

99.5% pure graphite (Item number: ES 350 F5) was purchased from Samjung (C & G, Gyeongsan, Korea), whereas reagent grade sulphuric acid (H_2_SO_4_), hydrochloric acid (HCl), sodium nitrate (NaNO_3_), hydrogen peroxide, potassium permanganate (KMnO_4_), DABCO, iron(III) acetate, palladium acetate and potassium dichromate (K_2_Cr_2_O_7_) were purchased from Sigma-Aldrich, Seoul, Korea, and they were used as received.

### 2.2. Methods

Microwave irradiation was carried out in a domestic microwave oven that was manufactured by Daewoo, Seoul, Korea, Model number: KR-B202WL, with an output power of 700 W operating at 2450 MHz. Field-emitting scanning electron microscopy (FE-SEM) was recorded on a Zeiss FEG-SEM Supra 25 (Seoul, Korea), and high resolution transmission electron microscopic (HRTEM) images, a high-angle annular dark-field (HAADF) and elemental energy dispersive spectroscopy (EDS) maps were recorded on TALOS F200X (Thermo Fisher Scientific Korea Ltd., Seoul, Korea) operating at 10 kV and 200 kV, respectively. No metal coating was applied to the samples during FE-SEM testing since the synthesized Pd@NCNT-rGO and Fe@NCNT-CF showed excellent conductivity. All of the electrochemical measurements were conducted on a CHI 660 electrochemical station (CH Instruments, Inc. Austin, TX, USA) with a conventional three-electrode system. The electrode was first prepared by polishing GCE (glassy carbon electrode) carefully with 1.2, 0.6 and 0.03 μm alumina powder and washing with deionized water, followed by sonication in isopropyl alcohol and finally rinsing with distilled water successively after each step. Finally, the electrode was dried under a stream of nitrogen. For methanol oxidation reactions, 5 μL of Pd@NCNT-rGO or Pd/C catalyst methanolic solution was dropped on the surface of GCE and dried. The loading of Pd on the GCE was limited to 68.72 μg cm^−2^. Then, 5 μL of Nafion (0.2%) was gently dropped onto the surface of the active material-anchored GCE and dried before the electrochemical experiments.

### 2.3. In-Situ Synthesis of Graphene-CNT Hybrids

A one step microwave procedure is used to concomitantly reduce and grow nitrogen-doped carbon nanotubes anchored on reduced graphene oxide. Graphene oxide synthesized by a modified Hummer’s method, palladium acetate and DABCO were mixed in a mortar-pestle at a weight ratio of 1:0.4:4 and subsequently transferred into a glass tube, partially sealed with a lid and microwaved for 240 s at 700 W to yield a fluffy solid [***Caution***: These microwave reactions release large amounts of gases and must be carried out in a fume hood. Even though we can scale up this technique to the kilogram scale, subjecting microwave radiation of large reactant batches results in the release of obnoxious gases and explosions. Thus, proper care and precautions must be taken to avoid damage and injury]. The resultant powder was washed with copious amounts of deionized water to remove unreacted DABCO and finally dried in an oven at 100 °C for 60 min to obtain Pd@NCNT-rGO.

### 2.4. Chromium (Cr(VI)) Adsorption Studies

Aqueous solutions with increasing Cr(VI) concentrations of 20, 40, 60, 80 and 100 mg L^−1^ were prepared by dissolving different amounts of K_2_Cr_2_O_7_ in deionized water. The pH was adjusted to 4 by adding HCl. 1 g of Fe@NCNT-CF was added to 500 mL of the prepared Cr(VI) solution. In order to analyze the amount of Cr(VI) adsorbed, 2.5 mL was drawn out, to which 1 mL of diphenylcarbazide was added, and the resulting solution was adjusted to a pH of 2 by the addition of dilute H_2_SO_4_. The adsorbed capacity of Cr(VI) was monitored by the UV absorption.

## 3. Results and Discussion

**Figure 1:**
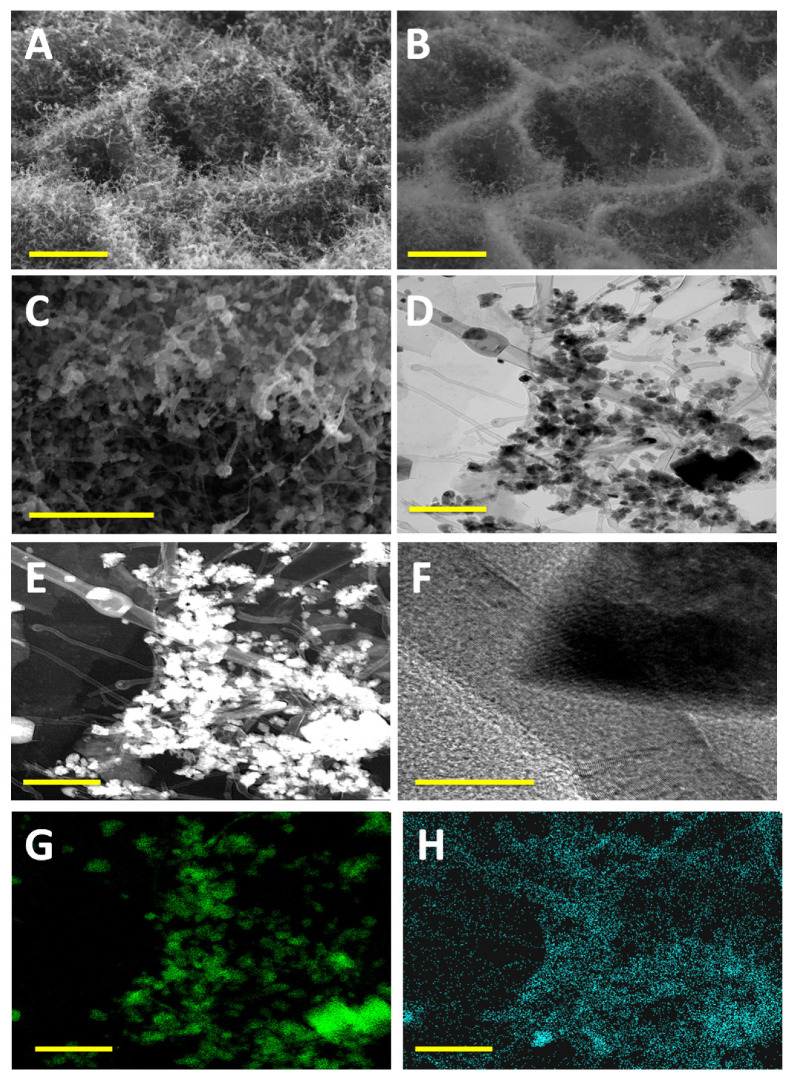
(**A**) Low-magnification SEM micrographs and (**B**) their corresponding secondary electron image; (**C**) high-magnification SEM of Pd@NCNT-rGO; (**D**) TEM image and (**E**) its corresponding high-angle annular dark-field (HAADF) image; (**F**) high-resolution TEM image of CNT and palladium nanoparticles; EDS maps of (**G**) palladium and (**H**) nitrogen. Scale bars are 2 µm in (**A**,**B**); 1 µm in (**C**); 500 nm in (**D**,**E**,**G**,**H**) and 50 nm in (**F**).

The microstructure and morphology of Pd@NCNT-rGO as studied by traditional ‘*in-lens*’ and back-scattered electron (BSE) SEM images is exhibited in Figure 1A–C, respectively. Both images show well-distributed, short carbon nanotubes grown on a reduced graphene oxide substrate. Additionally, the presence of palladium nanoparticles distributed along the walls of CNTs and on the surface of the reduced graphene oxide substrate can also be observed. This is further manifested in the BSE image (Figure 1B), wherein the palladium nanoparticles appear considerably brighter than both carbon nanotubes and the reduced graphene oxide substrate due to the fact that palladium nanoparticles backscatter electrons more strongly due to their higher atomic number when compared to the two carbon allotropes. A three-step procedure can be contemplated for the growth of carbon nanotubes from DABCO. In the first step, the DABCO moieties react with oxygen moieties of graphene oxide to yield reduced graphene oxide [18]. Additionally, this reduction reaction will be accelerated and augmented by the microwave irradiation, wherein the produced intense localized heat can effectively deoxygenize the graphene oxide. In the second step, the palladium nanoparticles generated by the disassociation of palladium acetate [19] are anchored on the defects of the graphene oxide generated during the synthesis of graphene oxide by Hummer’s method [20]. Thirdly, under intense heat produced by prolonged microwave radiation, DABCO thermally decomposes into nitrogen-rich hydrocarbons like H_2_NCH_2_CH_2_NH_2_ [21], which are captured by the palladium nanoparticles and, due to their inherent excellent dehydrogenation activity, are transformed into nitrogen-doped carbon nanotubes by the vapor–liquid–solid (VLS) growth mechanism of CNT [22].

The TEM image exhibited in Figure 1D and its corresponding dark field image in Figure 1E show the extensive presence of palladium particles both inside the carbon nanotubes and along their walls. The high-resolution TEM image (Figure 1F) shows that the nanotubes are multiwalled and are relatively straight with 40–45 walls, of which the outer 4–5 walls are defect-rich and the inner walls are fairly defect-free. Figure 1G,H shows the elemental EDS area maps of palladium and nitrogen, respectively, and the majority of nitrogen moieties are seen to be distributed along the walls of the nanotubes, with some minor portion on the surface of rGO, whereas in case of palladium they are well distributed as nanoparticles both on the rGO substrate and on and inside CNT.

**Figure 2:**
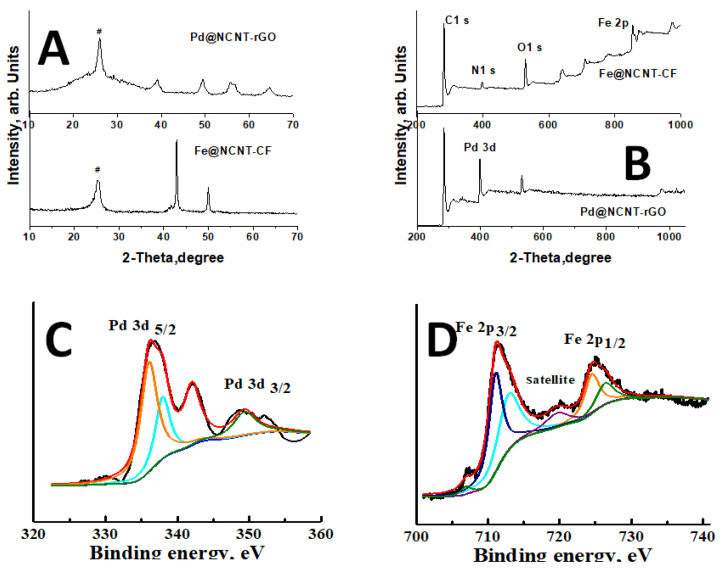
(**A**) XRD and (**B**) XPS plots of Pd@NCNT-rGO and Fe@NCNT-CF, respectively. Deconvoluted (**C**) Pd 3d and (**D**) Fe 2p spectra.

XRD was used to study the structure and chemical properties of Pd@NCNT-rGO and plotted in the top region of Figure 2A, which is dominated by a sharp peak at 26.01 (indicated by #), which can be attributed to the (002) peak of disordered carbon [23], whereas the relatively weak peaks at 34.51 and 64.69 can be attributed to (112) and (220) peaks of palladium nitride [24] and whereas the peaks at 42.4, 54.9 and 60.4 correspond to (110), (112) and (103) of the palladium oxide (JCPDS No.75-16210). In the case of Fe@NCNT-CF, the carbon peak is observed at 25.5 (highlighted by #), which indicates a higher degree of graphitization, whereas the two iron peaks at 42.9 and 47.91 correspond to iron nitride [25] and iron carbide/iron oxide [26], respectively. The deconvoluted XPS spectra of the Pd 3d plotted in Figure 2C show two major peaks [24] at 335.9 and 348.4 eV, corresponding to Pd 3d3/2 and Pd 3d5/2, regions, respectively. Besides these, two corresponding satellite peaks at 343.2 and 352.01 eV can also be observed. The dominant Pd 3d5/2 peak at 335.9 eV is characteristic of zero valent palladium, Pd(0) [27], whereas the peak at 373.2 eV indicates the formation of PdN [28] moieties. The deconvoluted XPS spectra of the Fe 2p binding energy region plotted in Figure 2B show two distinct peaks at 711.3 and 724.64 eV, corresponding to Fe 2p3/2 and Fe 2p1/2, respectively. Of the two peaks, the Fe 2p3/2 peak is sharper and can be further deconvoluted into two peaks at 711.3 and 713.12 eV, which can be attributed to iron nitride and iron hydroxides, whereas the Fe 2p1/2 peak is deconvoluted into two peaks at 724.5 and 726.6 eV corresponding to iron carbide [29] and iron nitride [30], respectively. In addition to these major peaks, minor but visually discernible peaks at 719.7 eV, which corresponds to iron nitride [31], and at 707.03, which corresponds to zero valent iron [32], can also be observed, indicating that the majority of iron is in the form of iron nitride with minor iron carbide impurities. A clearly distinguishable small peak observed at 719 eV is the satellite peak associated with Fe 2p3/2; in addition, there is a small weakly discernible satellite hump at 729.5 eV, which may be a satellite peak for Fe 2p1/2.

**Figure 3:**
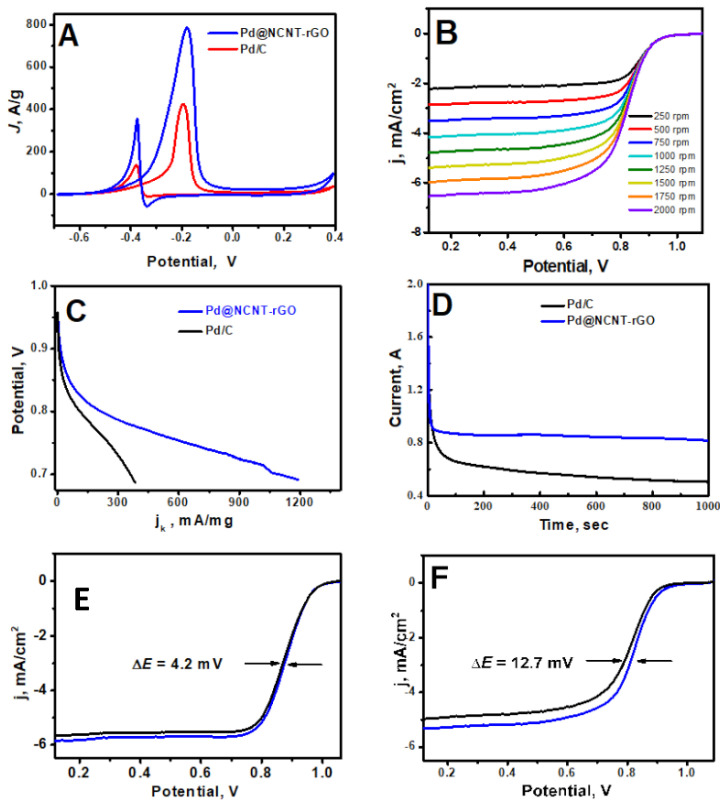
(**A**) CVs of Pd/C and Pd@NCNT-rGO in aqueous 1 M KOH + 1 M methanol solution at a scan rate of 50 mV/s. (**B**) ORR polarization curves of Pd@NCNT-rGO at different rotation rates. (**C**) Kinetic currents density (jk) at different potentials. (**D**) Current density–time curves. (**E**,**F**) The ORR polarization curves of Pd@NCNT-rGO and Pd/C before and after 1000 cycles, respectively.

The electro-catalytic activities of Pd/C and Pd@NCNT-rGO towards methanol oxidation were investigated by cyclic voltammetry (Figure 3A) in aqueous 1 M KOH + 1 M methanol solution at a scan rate of 50 mV/s (CV of GO in Figure S1 of supplementary file). Amongst the two Pd-based catalysts investigated, Pd@NCNT-rGO possesses a considerably higher activity and exhibits comparatively less anodic onset potential and a substantially higher current density. Specifically, the peak of the current density of the Pd@NCNT-rGO is 2.12 times higher than those of Pd/C. Pd@NCNT-rGO also possess a marginally lower onset potential at about −0.375 V, which is more negative than that of the Pd/C (−0.378 V).

The ratio of the forward (I_f_) to backward (I_b_) peak current (I_f_/I_b_) is indicative of the resistance of the catalyst to the accumulation of carbonaceous species (mostly carbonyls and carbon monoxide (CO)) onto the electrode surface [33]. When compared to Pd/C, the I_f_/I_b_ of Pd@NCNT-rGO is 2.47 times higher, which indicates that by embedding Pd nano-particles in a 3-D mesoporous carbonaceous nanostructure, not only can the catalytic activity be substantially improved but so can the tolerance of the catalyst towards CO poisoning, which can be attributed to two main reasons: the first is that the majority of palladium nanoparticles are embedded inside carbon nanotubes or on the walls of NCNT in protective carbon shells, which protects them from poisoning; and second, the unique 3-D mesoporous structure offers enough space to accommodate the generated carbonaceous species without affecting the catalytic activity. The enhanced electrocatalytic performance of Pd@NCNT-rGO over commercial Pd/C can be mainly attributed to two reasons: the presence of nitrogen moieties and well-dispersed palladium nanoparticles. First, the nitrogen moieties of carbon nanotubes synthesized from DABCO are themselves capable of showing a moderate electro-catalytic activity and promote a four-electron pathway for the oxygen reduction. Second, the presence of well-dispersed, nanosized palladium nanoparticles with exposed crystal facets (as can be observed in the HRTEM in Figure 1F) not only leads to a synergetic interaction between the carbonaceous support and palladium nanoparticles but also greatly enhances the adsorption binding energy of oxygen molecules on the exposed facets [34]. Figure 3B depicts RRDE (rotating ring-disk electrode) measurements at increasing rotation rates ranging from 250 to 2000 rpm for Pd@NCNT-rGO. A steady increase in the value of the absolute current density with an increasing rotation rate can be observed. The mass activity (MA) of Pd@NCNT-rGO and Pd/C catalysts was calculated by quantifying the mass current density (Figure 3C). When compared to Pd/C, our newly developed Pd@NCNT-rGO displays a considerably higher MA, which can be attributed to the well-dispersed Pd nanoparticles and also the presence of electro-active nitrogen moieties, due to nitrogen doping which can boost the catalytic activity. Chrono-amperometric tests (Figure 3D) were carried out to further evaluate the activity and stability of methanol electro-oxidation in a 1.0 M KOH solution containing 1.0 M methanol. In the whole measured time scale, Pd@NCNT-rGO shows a substantially higher current density when compared to Pd/C, reiterating that a better catalytic activity and stability can be achieved by embedding Pd nanoparticles in nitrogen-doped 3-D mesoporous rGO-NCNT structures. Stability is a vital criterion for the long-term applicability of an electro-catalyst, and the stability of our Pd@NCNT-rGO was studied via consecutive cycling for 1000 sweeps and was compared with that of Pd/C. As displayed in Figure 3E,F in which the blue line indicates first sweep and black line indicates 1000th sweep, the half-wave potential (E_1/2_) of the Pd@NCNT-rGO has a slight negative shift of 4.2 mV, while a negative shift of 12.7 mV is found for Pd/C, establishing the improved stability of the Pd@NCNT-rGO.

**Figure 4:**
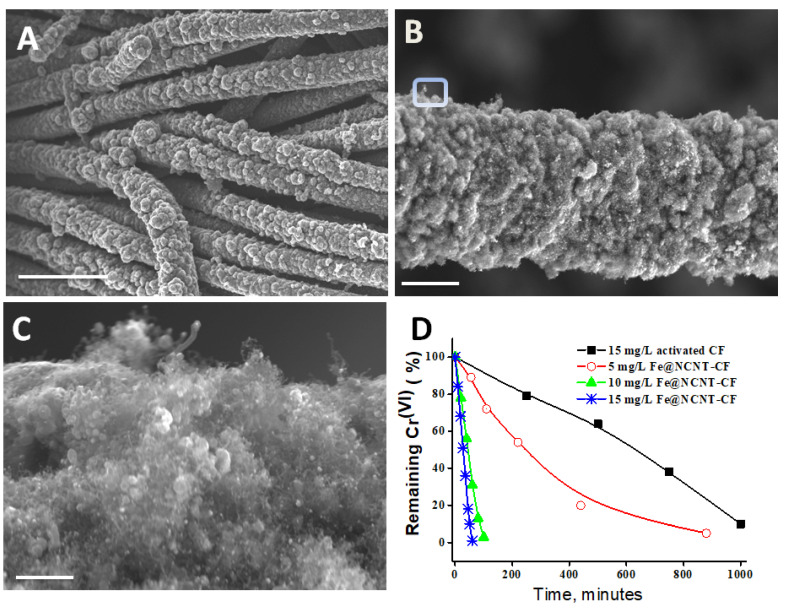
(**A**–**C**) Representative SEM micrographs at increasing magnifications of DABCO-derived nitrogen-doped carbon nanotubes grown on carbon fibers using an iron catalyst. Scale bars are (**A**) 25 µm; (**B**) 4 µm and (**C**) 500 nm. (**D**) Adsorption isotherms of hexavalent chromium on Fe@NCNT-CF nanostructures at various concentrations.

The utility of DABCO as a source of growth for nitrogen-doped CNT via microwave radiation was also investigated using iron as the catalyst and carbon fiber as the microwave-susceptible substrate. A synthesis procedure outlined in Section 2.3 was used with iron acetate acting as the source of iron catalyst nanoparticles and carbon fiber (CF) as the substrate. The CFs were pretreated to remove ‘sizing’ material that is routinely applied to the surface of fibers during the manufacturing process to protect the filaments during handling, and they were processed via thorough washing with 10% HCl and DI water successively. Subsequently, the fibers were chopped and mixed with DABCO and iron acetate in a 1:4:0.4 ratio and were subjected to microwave irradiation for 180 s to yield iron-embedded nitrogen-doped CNT-decorated carbon fibers (Fe@NCNT-CF). SEM micrographs at increasing magnifications, exhibited in Figure 4A–C, show a very high density growth of carbon nanotubes, unlike our previous reports wherein precursors like niacin (Vitamin B_3_) [35] and waste polyethylene terephthalate (wPET) [36] resulted in a sparse growth of CNT on the CF surface. This result further reiterates our contention that DABCO is an excellent source of nitrogen-doped CNT.

The rate of adsorption of chromium at different Fe@NCNT-CF concentrations is plotted in Figure 4D, showing that at all measured concentrations our Fe@NCNT-CF nanostructures showed a higher adsorption rate. When compared with CF alone, the complete removal of chromium by our newly developed Fe@NCNT-CF could be achieved in less than 100 min, whereas it took 1000 min in the case of activated CF. This outstanding performance of chromium absorption by flexible Fe@NCNT-CF hybrid nanostructures can be attributed to a high mesoporosity and open pore network, which facilitate the efficient removal of chromium ions by the nitrogen doping-induced defects on the walls of CNTs, via the well-dispersed and distributed iron oxide nanoparticles embedded in the three dimensional mesoporous nanoarchitectures of NCNT-CF.

## 4. Conclusions

In conclusion, we have developed a fast and facile microwave method to effectively reduce and grow nitrogen carbon nanotubes on reduced graphene oxide via a single precursor, namely DABCO. Morphological studies by SEM and TEM showed 3D mesoporous nanoarchitectures of nanometer-thin, micrometer-long carbon nanotubes vertically anchored on a reduced graphene oxide substrate. When applied as ORR, the Pd@NCNT-rGO nanostructure exhibited an exceptionally good electro-catalytic performance even after prolonged cycling, emphasizing the strong synergy between the reduced graphene oxide substrate, nitrogen moieties of nitrogen-doped carbon nanotubes and palladium nanoparticles distributed in these 3D carbon nanoarchitectures. The utility of our new technique for the synthesis of carbon nanotubes on other microwave-susceptible surfaces like carbon fibers using traditional iron catalysts is also demonstrated. Despite the curved nature of carbon fibers, highly dense nitrogen-doped carbon nanotubes can be grown, resulting in three-dimensional Fe@NCNT-CF whose utility as flexible adsorbents for the removal of hexavalent chromium ions from contaminated waters is also demonstrated.

## Data Availability

All data is contained within the article.

## Supplementary Materials

The following are available online at https://www.mdpi.com/article/10.3390/ma14112871/s1, Figure S1: CV of GO electrode in 1 M KOH solution.
