# Supplementary material for: DABCO Derived Nitrogen-Doped Carbon Nanotubes for Oxygen Reduction Reaction (ORR) and Removal of Hexavalent Chromium from Contaminated Water
Source: Materials (Basel). 2021 May 27;14(11):2871. doi: 10.3390/ma14112871 (PMC8199063; doi:10.3390/ma14112871)
Supplement: Supplementary file 1 [file materials-14-02871-s001.zip › materials-1232326-supplementary.pdf]

materials

Supplementary Materials

DABCO Derived Nitrogen-Doped Carbon Nanotubes for Oxygen Reduction Reaction (ORR) and Removal of Hexavalent Chromium from Contaminated Water

Vadahanambi Sridhar [1,*] and Hyun Park [1,2,*]

[1] Global Core Research Centre for Ships and Offshore Plants (GCRC-SOP), Pusan National University; Busan 46241, Korea
[2] Department of Naval Architecture and Ocean Engineering, Pusan National University, Busan 46241, Korea
* Correspondence: sridhar@pusan.ac.kr (V.S.); hyunpark@pusan.ac.kr (H.P.); Tel.: +82-51-510-2730 (H.P.)

Citation: Sridhar, V.; Park, H. DABCO Derived Nitrogen Doped Carbon Nanotubes for Oxygen Reduction Reaction (ORR) and Removal of Hexavalent Chromium from Contaminated Water. *Materials* **2021**, *14*, 2871. https://doi.org/10.3390/ma14112871

Academic Editor: Julia A. Baimova

Received: 7 May 2021
Accepted: 24 May 2021
Published: 27 May 2021

Publisher's Note: MDPI stays neutral with regard to jurisdictional claims in published maps and institutional affiliations.

Copyright: © 2021 by the authors. Licensee MDPI, Basel, Switzerland. This article is an open access article distributed under the terms and conditions of the Creative Commons Attribution (CC BY) license (http://creativecommons.org/licenses/by/4.0/).

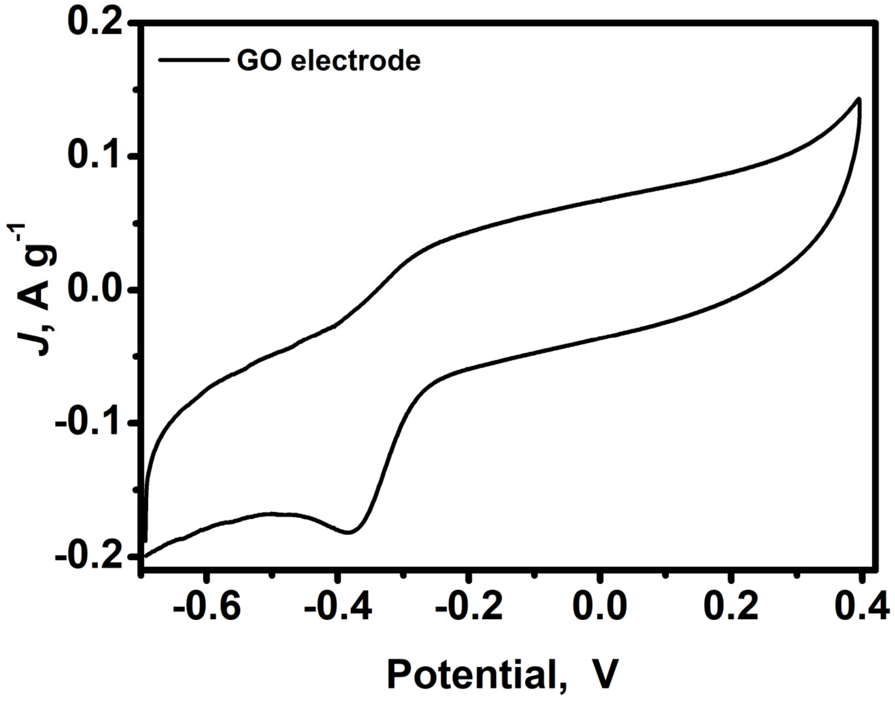

Figure S1. CV of GO electrode in 1 M KOH solution.
